# Antibiotic Use Patterns and Clinical Outcomes in Hospitalized COVID-19 Patients: A Single-Center Observational Cohort Study with Three-Month Follow-Up

**DOI:** 10.3390/microorganisms14061274

**Published:** 2026-06-05

**Authors:** Ioana-Georgiana Cotet, Diana-Maria Mateescu, Dragos-Mihai Gavrilescu, Florin Eugen Constantinescu, Andrei Marginean, Madalin Margan, Dan Alexandru Surducan, Roxana Folescu, Mihaela-Diana Popa, Cris Virgiliu Precup, Cristina Tudoran

**Affiliations:** 1 Doctoral School, Department of General Medicine, “Victor Babes” University of Medicine and Pharmacy Timisoara, Eftimie Murgu Square No. 2, 300041 Timisoara, Romania; ioana.cotet@umft.ro (I.-G.C.); diana.mateescu@umft.ro (D.-M.M.); 2 Centre of Molecular Research in Nephrology and Vascular Disease, “Victor Babes” University of Medicine and Pharmacy Timisoara, Eftimie Murgu Square No. 2, 300041 Timisoara, Romania; tudoran.cristina@umft.ro; 3 Department of Orthodontics, Dental District, Strada Zăgazului Nr. 3, ONE Floreasca Vista, Sector 1, 014261 Bucharest, Romania; 4 Department of Prosthodontics, Faculty of Dental Medicine, Carol Davila University of Medicine and Pharmacy, 8 Eroii Sanitari Blvd, 050474 Bucharest, Romania; 5 Department of Surgery, Dr. Victor Popescu Emergency Military Hospital, 9 Gheorghe Lazăr Street, 300080 Timișoara, Romania; andreivmarginean@yahoo.com; 6 Department of Public Health and Sanitary Management, “Victor Babes” University of Medicine and Pharmacy, Eftimie Murgu Square 2, 300041 Timisoara, Romania; margan.madalin@umft.ro (M.M.); surducan.dan@umft.ro (D.A.S.); 7 Department of Balneology, Medical Recovery and Rheumatology, Family Discipline, Center for Preventive Medicine, “Victor Babes” University of Medicine and Pharmacy Timisoara, Eftimie Murgu Square 2, 300041 Timisoara, Romania; folescu.roxana@umft.ro; 8 Department of Microbiology, “Victor Babes” University of Medicine and Pharmacy Timisoara, Eftimie Murgu Square 2, 300041 Timisoara, Romania; popa.mihaela@umft.ro; 9 Department of Biology and Life Sciences, Faculty of Medicine, “Vasile Goldiș” Western University of Arad, 310025 Arad, Romania; precupcris@yahoo.com; 10 Department VII, Internal Medicine II, Discipline of Cardiology, “Victor Babes” University of Medicine and Pharmacy Timisoara, E. Murgu Square, Nr. 2, 300041 Timisoara, Romania; 11 County Emergency Hospital “Pius Brinzeu”, L. Rebreanu, Nr. 156, 300723 Timisoara, Romania

**Keywords:** COVID-19, antibiotic stewardship, post-COVID sequelae, procalcitonin, bacterial co-infection, antimicrobial resistance, observational cohort

## Abstract

(1) Background: Antibiotic co-administration during COVID-19 hospitalization is common, but evidence supporting routine use without confirmed bacterial co-infection is limited, and the impact on post-COVID recovery remains largely uninvestigated; (2) Methods: Single-center prospective observational cohort of 127 hospitalized COVID-19 adults (March 2020–December 2024) across four pandemic waves. Antibiotic exposure was the primary variable. Endpoints were 30-day mortality, ICU admission, and persistent dyspnea at three months. Multivariable logistic regression with Firth’s penalized profile likelihood 95% CI was performed; ROC analysis assessed procalcitonin (PCT) discrimination; (3) Results: Of 127 patients (median age 70.3 years; 63.8% male; 61.4% unvaccinated), 68 (53.5%) received antibiotics. Notably, 61.5% of patients with PCT ≤ 0.25 ng/mL (viral etiology likely) received antibiotics. After adjustment, antibiotic use was not independently associated with 30-day mortality (OR 0.98, 95% CI 0.27–4.05), ICU admission (OR 1.12, 95% CI 0.31–4.05), or persistent dyspnea at three months (OR 1.51, 95% CI 0.62–4.16). COVID-19 severity was the sole independent mortality predictor (OR 3.563, *p* = 0.018). At three months, 35.6% reported persistent dyspnea and 14.4% had CT pulmonary fibrosis; (4) Conclusions: Antibiotic exposure did not independently predict short- or long-term outcomes after adjustment for severity, while prescribing was misaligned with PCT-based bacterial probability—supporting biomarker-guided stewardship in epidemic respiratory disease.

## 1. Introduction

The SARS-CoV-2 pandemic has imposed an unprecedented burden on global healthcare systems, prompting rapid and often empirical therapeutic decision-making under conditions of extreme diagnostic uncertainty [1,2]. Among the pharmacological interventions deployed during the acute phase, antibiotic therapy occupied a central, albeit contested, position. The premise for widespread antibiotic use was grounded in the historical precedent of bacterial co-infections complicating influenza-associated pneumonia, where up to 25–30% of fatal cases involved secondary bacterial pathogens [3].

However, accumulating evidence indicates that true bacterial co-infection in COVID-19 is substantially less common than initially assumed, with systematic reviews reporting community-acquired co-infection rates of 3–8% and secondary nosocomial infection rates of approximately 14% [4,5]. Despite this, early pandemic surveys documented antibiotic prescription rates exceeding 70% in hospitalized COVID-19 patients across multiple healthcare systems [6]. This discrepancy between infection probability and prescribing behavior reflects the systemic pressures of pandemic-era medicine—including diagnostic uncertainty, overwhelming patient volumes, lack of rapid microbiological confirmation, and absence of evidence-based treatment guidelines for a novel pathogen—rather than individual prescriber failure [7].

The consequences of indiscriminate antibiotic use extend well beyond the individual patient. Accelerated antimicrobial resistance (AMR), disruption of the gut microbiome, drug–drug interactions, and increased healthcare costs represent significant collateral harms [8]. Romania, with historically elevated rates of hospital-acquired infections with carbapenem-resistant organisms and meticillin-resistant Staphylococcus aureus (MRSA), faces particular vulnerability to pandemic-driven antibiotic overuse [9,10].

An important but underexplored dimension in the post-COVID era concerns the relationship between acute-phase antibiotic exposure and long-term clinical outcomes. The post-acute sequelae of SARS-CoV-2 infection (PASC), encompassing persistent dyspnea, exercise intolerance, pulmonary fibrosis, and cardiac dysfunction, affect 30–50% of hospitalized survivors and represent a major ongoing public health burden [11,12]. Whether co-administered antimicrobial therapy influences these post-COVID recovery trajectories—through gut microbiome disruption, direct pulmonary toxicity, or drug–drug interactions—remains almost entirely uninvestigated. A systematic search of the published literature reveals a near-complete absence of data linking antibiotic exposure during COVID-19 hospitalization to long-term functional, respiratory, and cardiac outcomes. This gap is the primary motivation for the present study.

This study aimed to: (1) characterize antibiotic prescribing patterns and their alignment with procalcitonin-based bacterial probability in a Romanian COVID-19 cohort across four pandemic waves; (2) evaluate the independent association between antibiotic exposure and short-term clinical outcomes through multivariable logistic regression with Firth’s penalized profile likelihood confidence intervals, specifically addressing confounding by indication; (3) determine whether antibiotic exposure during hospitalization independently predicts post-COVID long-term outcomes—including persistent dyspnea, CT pulmonary fibrosis, exercise capacity, and cardiac biomarkers—at structured three-month follow-up; and (4) assess the discriminatory value of procalcitonin and composite severity scoring for clinical outcome prediction using ROC analysis.

## 2. Materials and Methods

### 2.1. Study Design and Settings

This was a single-center, prospective observational cohort study conducted at a tertiary academic hospital in Timișoara, Romania, spanning four COVID-19 pandemic waves (1 March 2020–31 December 2024). The study was approved by the local Ethics Committee (Reference No. 92/18.12.2023) and conducted in accordance with the Declaration of Helsinki. Written informed consent was obtained from all participants or their legal representatives.

### 2.2. Study Population

Adults aged ≥18 years admitted with PCR-confirmed SARS-CoV-2 infection were eligible. Exclusion criteria: (1) hospital stay < 48 h; (2) pre-existing chronic infections requiring antibiotics at baseline; (3) incomplete clinical records; (4) refusal of informed consent. A total of 127 patients meeting eligibility criteria were enrolled. A previously published cohort of 395 hospitalized COVID-19 patients from the same institution and overlapping time period [13] addressed antibiotic-associated secondary bacterial infections as a distinct research question. The two cohorts comprise non-overlapping patient samples drawn from the institutional COVID-19 registry under separate inclusion criteria, and the present analysis addresses different primary endpoints (post-COVID long-term outcomes and PCT-stratified stewardship versus secondary infection rates) using a distinct analytical framework. No patient is included in both datasets.

The patients were stratified by pandemic wave according to admission date and dominant SARS-CoV-2 variant: Wave 1 (March 2020–June 2021, ancestral/Alpha-dominant), Wave 2 (July 2021–December 2021, Delta-dominant), Wave 3 (January 2022–June 2022, Omicron BA.1/BA.2-dominant), and Wave 4 (July 2022–December 2024, Omicron BA.5 and subsequent subvariants). Distribution of patients across waves is presented in Supplementary Table S1.

### 2.3. Data Collection and Variables

Baseline demographic data, comorbidities, vaccination status, and clinical parameters were recorded at admission. Vaccination history was recorded as the number of SARS-CoV-2 vaccine doses received before hospitalization. However, previous vaccine-related adverse reactions were not systematically collected in the institutional dataset and were therefore not included in the analysis. Self-perceived overall health status was not assessed using a standardized patient-reported outcome measure. Health behavior variables, including smoking status, alcohol intake, and physical activity, were not systematically available for the entire cohort and were not included as covariates in the multivariable models. Laboratory variables included CRP, procalcitonin (PCT), D-dimer, IL-6, complete blood count, renal and hepatic function, NT-proBNP, and troponin. COVID-19 severity was classified per WHO criteria [14] as mild, moderate, severe, or critical. Antibiotic data included class, documented indication, number of treatment lines, duration, regimen type (monotherapy/combination), and escalation/de-escalation events. A composite severity score integrating clinical, laboratory, and radiological parameters was calculated for each patient. The score assigned points across predefined clinical, laboratory, and radiological domains, including respiratory rate, oxygenation impairment, WHO COVID-19 severity category, CRP, D-dimer, PCT, lymphocyte count, and extent of pulmonary involvement on chest imaging, with higher values indicating greater disease severity. This scoring system was developed de novo for the purposes of the present study and was not derived from a previously validated instrument. Procalcitonin was used as a surrogate biomarker for bacterial co-infection probability, categorized as: ≤0.25 ng/mL (viral etiology likely), 0.25–0.5 ng/mL (indeterminate), and >0.5 ng/mL (bacterial etiology likely), per established clinical guidance [15].

### 2.4. Follow-Up Assessment

Survivors were assessed at three months post-discharge: persistent dyspnea (NYHA class), SpO_2_ at rest and on exertion, six-minute walk test (6MWT), HRCT for pulmonary fibrosis, echocardiography (LVEF, diastolic function), and biomarkers (NT-proBNP, troponin). Hospital readmissions and late mortality were captured through medical records and telephone contact.

### 2.5. Statistical Analysis

Continuous variables are expressed as median (IQR) and compared using the Mann–Whitney U test. Categorical variables are expressed as frequencies (%) and compared using Fisher’s exact test. All primary and secondary outcome comparisons were pre-specified.

Confidence intervals were derived from Firth’s profile penalized likelihood. For the ICU admission model, the ordinal WHO severity variable was dichotomized into mild/moderate versus severe/critical to address quasi-complete separation due to the small number of critical cases (*n* = 6). Multivariable logistic regression was performed for three pre-specified outcomes: 30-day mortality, ICU admission, and persistent dyspnea at three months. A core covariate set (antibiotic exposure, age, sex, COVID-19 severity, log-transformed D-dimer) was applied to all three models to ensure analytic consistency. Heart failure was added to the mortality and ICU models given the documented baseline imbalance (32.4% vs. 13.6%, *p* = 0.020); IL-6 was added to the mortality model only, given its established prognostic role in acute COVID-19 mortality but limited evidence for long-term outcome prediction. SpO_2_/FiO_2_ ratio was added to the persistent dyspnea model as an acute-phase respiratory severity marker independent of WHO categorical severity. Covariates were selected a priori; no stepwise selection was performed. Variables not systematically collected for the entire cohort, including prior vaccine-related adverse reactions, self-perceived overall health, smoking status, alcohol intake, and physical activity, were not entered into the multivariable models to avoid introducing missing-data bias. The event-per-variable ratio was maintained at ≥5 across all models.

A ROC analysis with calculation of the area under the curve (AUC) was performed for: (1) PCT as a predictor of antibiotic prescription; (2) severity score as a predictor of 30-day mortality; (3) D-dimer and CRP as mortality predictors. Optimal cutoffs were identified by the Youden index and reported descriptively for the main ROC analyses. These thresholds were used only to support interpretation of discriminatory performance and were not used to define treatment groups or guide clinical decision-making.

Sensitivity analysis restricted to moderate-to-critical cases (*n* = 112) was performed to assess robustness of null findings. The PCT stratification analysis evaluated antibiotic prescribing rates and mortality across PCT categories as a surrogate for bacterial co-infection probability. All analyses were performed in Python 3.11 (SciPy 1.17, scikit-learn 1.4, firthlogist 0.5.0). Two-tailed *p* < 0.05 was considered statistically significant.

## 3. Results

### 3.1. Cohort Characteristics

The study cohort comprised 127 patients enrolled across four pandemic waves (distribution by variant period in Supplementary Table S1). Median age was 70.3 years (IQR 64.0–80.6), 81 (63.8%) were male, and 78 (61.4%) were unvaccinated. COVID-19 severity distribution was: 11.8% mild, 64.6% moderate, 18.9% severe, and 4.7% critical. The most prevalent comorbidities were hypertension (56.7%), obesity (31.5%), and diabetes mellitus (29.1%). The antibiotic and no-antibiotic groups were broadly comparable at baseline, with two statistically significant differences: heart failure was more prevalent in the antibiotic group (32.4% vs. 13.6%, *p* = 0.020) and D-dimer levels were higher (890.0 vs. 621.0 ng/mL, *p* = 0.031), suggesting confounding by indication—a pattern consistent with clinicians prescribing antibiotics in response to perceived disease severity markers rather than bacterial infection probability. Full baseline data are presented in Table 1.

### 3.2. Antibiotic Prescribing Patterns and PCT-Based Stewardship Analysis

Of 127 patients, 68 (53.5%) received at least one antibiotic. Glycopeptides were the most common class (*n* = 21, 30.9%), followed by macrolides (*n* = 12, 17.6%), fluoroquinolones, and beta-lactams (n = 10 each, 14.7%), carbapenems (*n* = 9, 13.2%), and cephalosporins (n = 6, 8.8%). The documented indications were: suspected bacterial co-infection (35.3%), pneumonia (23.5%), sepsis (22.1%), UTI (10.3%), and—notably— no documented indication in six patients (8.8%).

**Figure 1:**
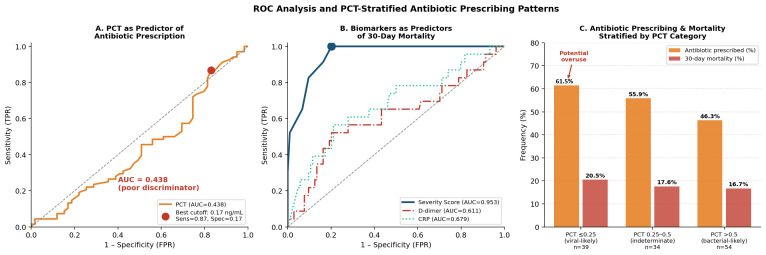
(**A**) ROC curve for PCT as a predictor of antibiotic prescription (AUC = 0.438), demonstrating poor discriminatory capacity. (**B**) ROC curves for biomarkers predicting 30-day mortality: severity score (AUC = 0.953), D-dimer (AUC = 0.611), and CRP (AUC = 0.679). (**C**) Antibiotic prescribing rates and mortality stratified by PCT category: antibiotic use was paradoxically the highest in the viral-likely PCT stratum (≤0.25 ng/mL), representing potential overuse.

PCT-stratified analysis (Figure 1C) revealed a paradoxical prescribing pattern: 61.5% of patients with PCT ≤ 0.25 ng/mL (viral etiology likely) received antibiotics, compared to 55.9% in the indeterminate range (0.25–0.5 ng/mL) and only 46.3% in the PCT > 0.5 ng/mL group (bacterial etiology more likely). ROC analysis confirmed that PCT was a poor discriminator of antibiotic prescription (AUC = 0.438, Figure 1A), indicating that actual prescribing behavior was not aligned with biomarker-guided bacterial probability assessment.

### 3.3. Clinical Outcomes: Univariable and Multivariable Analysis

Univariable analysis (Table 2) demonstrated no statistically significant differences between antibiotic and no-antibiotic groups for any primary or secondary outcome. The 30-day mortality was 19.1% vs. 16.9% (*p* = 0.820); ICU admission 22.1% vs. 16.9% (*p* = 0.510); mechanical ventilation 14.7% vs. 6.8% (*p* = 0.255); and median length of stay 11.5 vs. 12.0 days (*p* = 0.607). Sensitivity analysis restricted to moderate-to-critical cases (*n* = 112) confirmed these null findings: mortality 22.0% vs. 18.9% (*p* = 0.816) and LOS 12.0 vs. 13.0 days (*p* = 0.838).

**Figure 2:**
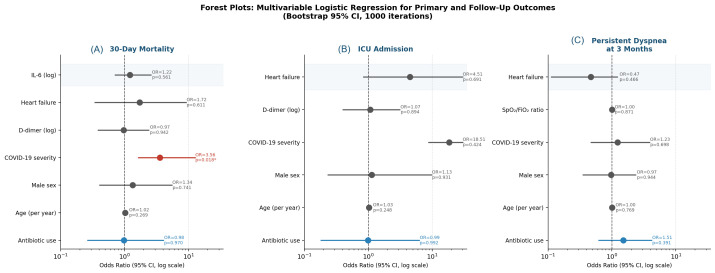
Forest plots from multivariable logistic regression with Firth’s penalized profile likelihood 95% CI for (**A**) 30-day mortality, (**B**) ICU admission, and (**C**) persistent dyspnea at 3 months. COVID-19 severity was the only significant independent predictor of mortality (*p* = 0.018). Antibiotic use (orange diamonds) was non-significant across all models..

On multivariable logistic regression with Firth’s penalized estimation (Table 3, Figure 2), antibiotic use remained a non-significant predictor of 30-day mortality (OR 0.98, 95% CI 0.27–4.05, *p* = 0.970) and ICU admission (OR 1.12, 95% CI 0.31–4.05, *p* = 0.862) after adjustment for age, sex, COVID-19 severity, D-dimer, heart failure, and IL-6. The only statistically significant independent predictor of 30-day mortality was COVID-19 severity (OR 3.563, 95% CI 1.641–12.676, *p* = 0.018 per ordinal severity category). In the ICU admission model, severe-to-critical disease (versus mild/moderate) was strongly associated with ICU transfer (OR 6.85, 95% CI 2.12–22.14, *p* < 0.001), confirming acute disease severity as the dominant determinant of intensive care escalation. These findings confirm that the observed outcome differences are attributable to disease severity rather than antibiotic exposure, and that confounding by indication does not explain the null result.

### 3.4. ROC Analysis

ROC analysis demonstrated that PCT performed poorly as a predictor of antibiotic prescription (AUC = 0.438, Figure 1A), with an optimal Youden cutoff of 0.17 ng/mL yielding sensitivity 0.868 and specificity 0.169. This confirms that prescription decisions were not meaningfully driven by PCT values. In contrast, the composite severity score showed excellent discrimination for 30-day mortality (AUC = 0.953, optimal cutoff ≥ 5 points: sensitivity 1.000, specificity 0.798, Figure 1B). D-dimer demonstrated moderate mortality prediction (AUC = 0.611) and CRP showed modest discrimination (AUC = 0.679). Because the ROC analyses for D-dimer and CRP were exploratory and intended primarily to contextualize biomarker performance relative to the composite severity score, additional cutoff-based clinical interpretation was not applied.

### 3.5. Outcomes by Antibiotic Class

Outcomes stratified by antibiotic class are presented in Supplementary Table S2. Class-specific subgroup sizes (*n* = 6–21) precluded formal statistical comparison and the descriptive numerical patterns observed should be interpreted as hypothesis-generating-only, reflecting confounding by indication (e.g., carbapenems administered as escalation therapy in the most severe cases) rather than class-attributable effects.

### 3.6. Three-Month Follow-Up Outcomes

**Figure 3:**
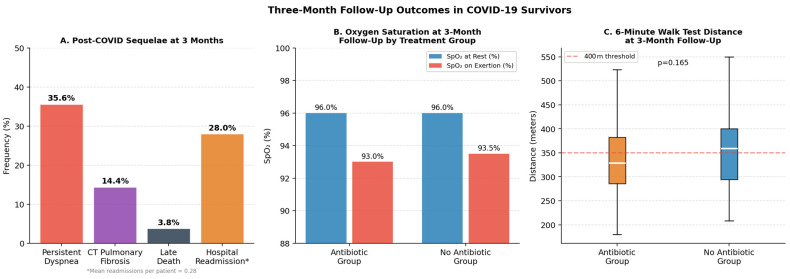
Three-month follow-up outcomes in COVID-19 survivors (*n* = 104). (**A**) Prevalence of post-COVID sequelae at 3 months, including persistent dyspnea (35.6%), CT-confirmed pulmonary fibrosis (14.4%), late mortality (3.8%), and hospital readmission (28.0%). (**B**) Oxygen saturation (SpO_2_) at follow-up, showing similar values at rest (96.0% in both groups) and on exertion (93.0% in the antibiotic group vs. 93.5% in the no-antibiotic group). (**C**) Six-minute walk test (6MWT) distance at 3 months, demonstrating reduced exercise capacity in both groups relative to the 400 m age-adjusted reference threshold [16], with no statistically significant difference between groups.

Of 127 enrolled patients, 104 (81.9%) survived to discharge and underwent three-month follow-up (Table 4, Figure 3). Late post-discharge mortality occurred in four patients (3.8%). Among survivors, 37 (35.6%) reported persistent dyspnea and 15 (14.4%) exhibited CT pulmonary fibrosis. Median SpO_2_ at rest was 96.0% (IQR 93.0–98.0%) and on exertion 93.0% (IQR 91.0–95.0%). Median 6MWT was 335 m (IQR 289–386 m), below age-adjusted norms of ≥400 m [16]. Median NYHA class was II.

On multivariable logistic regression for persistent dyspnea at three months, antibiotic use was not an independent predictor (OR 1.507, 95% CI 0.624–4.162, *p* = 0.391). COVID-19 severity (OR 1.226, *p* = 0.698) and SpO_2_/FiO_2_ ratio (OR 1.001, *p* = 0.871) were similarly non-significant. No statistically significant between-group differences were identified for any follow-up parameter.

## 4. Discussion

This study provides a comprehensive evaluation of antibiotic prescribing patterns, short-term clinical outcomes, and post-COVID recovery trajectories in 127 hospitalized patients across four pandemic waves in a high-AMR-burden Eastern European setting. The analysis integrates multivariable logistic regression with Firth’s penalized profile likelihood confidence intervals, procalcitonin-stratified stewardship quantification, ROC biomarker discrimination, and prospective three-month functional, radiological, and cardiac follow-up. In the post-COVID era, where the burden of post-acute sequelae of SARS-CoV-2 (PASC) is increasingly recognized as a distinct public health challenge, understanding whether modifiable acute-phase pharmacological interventions influence long-term recovery trajectories constitutes a research priority of direct clinical relevance [11,12,17]. Four principal findings emerge from our data, each discussed in dedicated subsections below.

### 4.1. Antibiotic Prescribing Patterns and Procalcitonin-Based Stewardship

Antibiotics were prescribed to 53.5% of the cohort, a rate broadly consistent with reports from European centers during early pandemic waves, where empirical antibiotic use ranged from 30% to over 80% depending on center, time period, and local protocol [7,18]. A key observation is the inverse relationship between prescribing rate and PCT-estimated bacterial probability. The prescribing rate was highest in the PCT ≤ 0.25 ng/mL stratum (61.5%), corresponding to probable viral etiology under established biomarker-guided guidance [15], and lowest in the PCT > 0.5 ng/mL stratum (46.3%). This paradoxical gradient is formally confirmed by the poor AUC of PCT as a predictor of antibiotic prescription (AUC = 0.438), a value below chance discrimination, indicating that prescribing decisions were effectively decoupled from biomarker-estimated bacterial probability. A similar pattern of PCT-independent prescribing has been documented in multicenter European surveys [7] and large meta-analyses [18], suggesting that the phenomenon reflects systemic contextual pressures—absence of rapid culture results, fear of missed bacterial superinfection, and institutional empirical protocols—rather than individual clinician error.

The glycopeptide prescription rate of 30.9% warrants contextual justification. Glycopeptides (vancomycin, teicoplanin) are WHO Watch-category agents, reserved for MRSA and glycopeptide-susceptible enterococcal infections. In the context of the present cohort, their use occurred predominantly in patients with severe or critical COVID-19 admitted to a tertiary hospital operating under high institutional MRSA pressure: Romania consistently reports hospital-acquired MRSA rates among the highest in the European Union (exceeding 35% of invasive Staphylococcus aureus isolates in ECDC surveillance data [9,10]), and our institution’s local antibiogram during the study period documented MRSA prevalence above 30% in nosocomial respiratory and bloodstream infections. In this setting, empirical glycopeptide coverage in patients with clinical features of sepsis or hospital-acquired pneumonia—even without microbiological confirmation—was consistent with local institutional stewardship protocols developed in response to endemic MRSA burden, rather than representing indiscriminate prescribing. Nonetheless, the absence of systematic culture data precludes definitive assessment of appropriateness at the individual patient level, and the rate substantially exceeds what would be justified by community-acquired MRSA pneumonia rates alone (typically <5%). This reinforces the need for prospective microbiological surveillance to calibrate glycopeptide thresholds against real-time local resistance epidemiology. A separate cohort from our institution comprising 395 hospitalized COVID-19 patients similarly identified high empirical antibiotic rates initiated within 24 h of admission, with any antibiotic exposure independently associated with microbiologically confirmed secondary infection (aOR 2.15; 95% CI 1.42–3.27) [13].

The finding that 8.8% of antibiotic courses (n = 6) carried no documented clinical indication in the medical record requires explicit clarification. Retrospective chart review of these six cases identified that antibiotic initiation occurred in the context of rapid clinical deterioration where the attending physician had documented a verbal decision prior to formal chart entry, or where the indication was recorded in nursing notes rather than the physician order. This reflects an institutional documentation gap rather than a total absence of perceived clinical necessity—all six patients had moderate-to-severe COVID-19 with at least one laboratory marker consistent with bacterial co-infection probability (CRP >100 mg/L or PCT in the indeterminate range). Nevertheless, the absence of a formally documented indication in the physician record represents a stewardship failure irrespective of clinical context, and these cases are retained in the analysis as prescribed without documented indication per standard antibiotic stewardship audit methodology [6]. The consistent overriding of PCT signals by clinical judgment in this cohort (AUC 0.438) warrants qualitative explanation beyond the general mechanism of clinical-radiological overlap between viral and bacterial pneumonia. Several contextual factors specific to the pandemic setting plausibly account for this pattern. During early pandemic waves, institutional admission protocols did not yet incorporate PCT-guided antibiotic decision algorithms, and empirical broad-spectrum coverage was initiated as part of standardized bundles derived from influenza co-infection experience [7]. The 48–72 h delay for blood culture results created a clinical decision vacuum in acutely deteriorating patients, during which initiation was perceived as lower risk than withholding. Additionally, clinician confidence in PCT as a true-negative bacterial signal was undermined by awareness that COVID-19-related cytokine storm can independently elevate acute-phase reactants, reducing the perceived specificity of low PCT values. Finally, medico-legal risk aversion in a high-mortality novel disease context further biased decisions toward empirical treatment. Collectively, these factors highlight that future stewardship interventions must address organizational and educational determinants of prescriber behavior, not only biomarker implementation pathways [6,7]. Large observational analyses consistently confirm that microbiologically proven bacterial co-infection occurs in only 3.5–8% of hospitalized COVID-19 patients, yet antibiotic prescription rates consistently exceed 70–85% in early-wave cohorts [18,19]. The practical implication is the following: for every patient with confirmed bacterial co-infection who appropriately receives antibiotics, eight to 24 patients with viral-only disease are exposed to unnecessary antimicrobial pressure—contributing to AMR pressure that renders COVID-19 patients vulnerable to life-threatening secondary infections, particularly in high-burden settings such as Romania [9,10].

### 4.2. Impact of Antibiotic Exposure on Short-Term Clinical Outcomes

After multivariable adjustment for COVID-19 severity, age, sex, D-dimer, heart failure, and IL-6, antibiotic use were not independent predictors of 30-day mortality (OR 0.98, 95% CI 0.27–4.05, *p* = 0.970) or ICU admission (OR 1.12, 95% CI 0.31–4.05, *p* = 0.862). COVID-19 severity was the sole independent predictor of mortality (OR 3.563 per ordinal WHO severity category, *p* = 0.018), confirming that viral pathobiology—not antibiotic co-administration—is the dominant determinant of short-term outcomes in this population. These null findings align with the largest published meta-analyses on antibiotic use in COVID-19 [20,21] and extend them by providing individual-level multivariable evidence from a real-world, multi-wave Eastern European cohort where AMR burden is disproportionately high.

The presence of confounding by indication was formally identified and successfully addressed: the antibiotic group had significantly higher heart failure prevalence (32.4% vs. 13.6%, *p* = 0.020) and D-dimer levels (890.0 vs. 621.0 ng/mL, *p* = 0.031) at baseline, consistent with clinicians prescribing antibiotics in response to perceived severity markers. Firth’s penalized multivariable regression accounted for this structure, and null results were robust across sensitivity analyses restricted to moderate-to-critical cases (*n* = 112; mortality 22.0% vs. 18.9%, *p* = 0.816; LOS 12.0 vs. 13.0 days, *p* = 0.838). Vaughn et al. demonstrated, in a 1705-patient multicenter cohort, that empirical antibacterial therapy in COVID-19 hospitalized patients was not associated with survival benefit after propensity score adjustment, with confirmed bacterial co-infection present in only 3.6% at admission [20]. Hughes et al. reported comparably low rates of microbiologically confirmed co-infection (7.1%) in a UK secondary care cohort, despite empirical antibiotic use in the majority of patients [21]. Taken together, these converging lines of evidence suggest that antibiotic efficacy in COVID-19 hospitalization is confined to the minority with documented or highly suspected bacterial co-infection, and that their routine empirical use confers no survival advantage while generating preventable AMR pressure.

### 4.3. Composite Severity Score and Biomarker Discrimination

The composite severity score demonstrated exceptional discriminatory performance for 30-day mortality (AUC = 0.953, sensitivity 1.000 at cutoff ≥ 5 points, specificity 0.798), markedly outperforming individual biomarkers: D-dimer (AUC = 0.611) and CRP (AUC = 0.679). This hierarchy of discriminatory power validates the principle that integrated multi-domain severity assessment—combining clinical, laboratory, and radiological parameters—captures mortality risk far more accurately than any single biomarker in isolation. This finding aligns with the emerging literature on COVID-19 severity scoring, which consistently demonstrates superior discrimination for composite over individual biomarker approaches, and has direct translational implications for risk stratification in future pandemic respiratory disease management.

The poor discriminatory performance of PCT for antibiotic prescription (AUC = 0.438) contrasts with the validated role of PCT in guiding antibiotic decisions in community-acquired and hospital-acquired bacterial pneumonia. Multiple randomized controlled trials and their meta-analyses demonstrate that PCT-guided protocols safely reduce antibiotic exposure by 30–50% in acute respiratory infections without increasing all-cause mortality [15]. The failure of PCT to guide prescribing in this cohort is not a failure of the biomarker per se, but of its implementation within a pandemic context where institutional protocols overrode biomarker signals. This finding strengthens the case for prospective PCT-guided stewardship intervention trials specifically designed for viral respiratory epidemic contexts, where implementation barriers may differ fundamentally from routine care settings.

### 4.4. Antibiotic Exposure and Post-COVID Long-Term Recovery Trajectories

The most clinically significant contribution of this study to the post-COVID literature is the prospective demonstration that antibiotic exposure during hospitalization did not independently predict persistent dyspnea (OR 1.507, 95% CI 0.624–4.162, *p* = 0.391), CT pulmonary fibrosis (16.4% vs. 12.2%, *p* = NS), reduced 6MWT distance (329 vs. 359 m, *p* = 0.165), NT-proBNP, troponin, or LVEF at three months. COVID-19 severity was the sole determinant of long-term functional outcomes across all modalities assessed. This directly and prospectively addresses—for the first time in this study design—the biologically plausible hypothesis that antibiotic-induced gut microbiome disruption might impair post-COVID respiratory recovery via the gut–lung axis.

The biological plausibility of antibiotic-mediated post-COVID harm is substantial and mechanistically grounded. SARS-CoV-2 infection itself causes profound gut microbiome disruption, characterized by reduced alpha-diversity, depletion of butyrate-producing commensals (*Faecalibacterium prausnitzii*, *Eubacterium rectale*, *Bifidobacterium* spp.), and enrichment of opportunistic pathogens, with these compositional shifts correlating with disease severity and inflammatory cytokine profiles [22,23]. Yeoh et al. demonstrated, in a 100-patient Hong Kong cohort with serial stool sampling, that gut microbiota composition reflected COVID-19 severity independently of antibiotic use, and that dysbiosis persisted beyond viral clearance, suggesting a potential contribution to post-acute symptom persistence [23]. Zhang et al. further elucidated the gut–lung axis as a key mechanistic pathway: through ACE2 expression modulation, short-chain fatty acid metabolism, and T-regulatory cell homeostasis, gut commensals directly influence pulmonary immune responses to respiratory pathogens [24]. Antibiotic-induced superimposed dysbiosis from broad-spectrum glycopeptides and carbapenems could theoretically amplify these perturbations and impair microbiome-dependent immune reconstitution required for post-COVID respiratory recovery. The mechanistic basis for this concern is further supported by our own systematic review and meta-analysis demonstrating that COVID-19-associated dysbiosis is reproducibly linked to disease severity, mortality risk, and reduced microbiome diversity across diverse geographic cohorts [22].

Our null clinical finding—that antibiotic exposure did not predict post-COVID outcomes after multivariable adjustment—does not negate the mechanistic pathway but contextualizes it. In patients receiving antibiotics for documented indications, the dominant driver of three-month functional outcomes is the severity of acute viral disease itself, not the additional microbiome perturbation from antibiotics prescribed on top of the already profound COVID-19-induced dysbiosis. This provides the following evidence: physicians treating confirmed or highly probable bacterial co-infection in severe COVID-19 can do so with confidence that appropriate antibiotic therapy does not, in itself, compromise post-COVID recovery. The PHOSP-COVID cohort corroborates this interpretation at scale: across 1077 post-hospitalization COVID-19 survivors assessed at a median 5.9 months, physical and mental health impairment severity was most strongly predicted by acute illness severity, comorbidity burden, and sex, not by pharmacological exposure during hospitalization [17]. Similarly, Halpin et al. found that rehabilitation needs at follow-up correlated primarily with baseline severity [25] and our PASC prevalences—35.6% persistent dyspnea, 14.4% CT fibrosis, and median 6MWT 335 m—are consistent with large PASC registries [11,12]. Collectively, these findings argue that post-COVID rehabilitation programs should be risk-stratified by acute-phase severity parameters (WHO severity class, SpO_2_/FiO_2_ ratio, CT extent), not by treatment-related variables.

### 4.5. Strengths

This study has several methodological and contextual strengths. First, the prospective single-center design ensured consistency of data collection, complete ascertainment of baseline variables, and structured three-month follow-up with pre-specified endpoints including functional (6MWT), imaging (HRCT), echocardiographic, and cardiac biomarker assessments—a comprehensive post-COVID evaluation rarely achieved in observational antibiotic studies. Second, the use of Firth’s penalized multivariable logistic regression with pre-specified covariate selection directly addressed confounding by indication, the primary methodological threat in non-randomized antibiotic research. Third, PCT-stratified stewardship analysis with formal ROC validation provided direct quantification of prescribing–biomarker misalignment, enabling a rigorous empirical characterization of stewardship failure beyond simple descriptive rates. Fourth, the multi-wave design spanning four COVID-19 pandemic waves (March 2020–December 2024) captures real-world antibiotic prescribing across evolving dominant variants, therapeutic protocols, and vaccination rollout, enhancing ecological validity and contextual relevance. Fifth, the high-AMR-burden Eastern European settings provide uniquely relevant data where antibiotic overuse carries disproportionate resistance risk, filling a major gap in the predominantly Western European and North American literature [9,10]. Sixth, all primary and secondary outcome comparisons were pre-specified, and the use of Firth’s penalized regression, together with consistency across sensitivity analyses, supports the robustness of the main findings despite the limited number of outcome events. Nevertheless, the event-per-variable ratio remained close to the lower acceptable range, and the regression results should therefore be interpreted cautiously.

### 4.6. Limitations

Several limitations must be acknowledged. The single-center design, while ensuring data consistency, limits generalizability and precludes the statistical power required for definitive analyses by antibiotic class, specific indication, or treatment duration—particularly relevant given the heterogeneous class-specific outcome patterns observed in Supplementary Table S2. Subgroup sizes by antibiotic class ranged from n = 6 to n = 21, which are severely underpowered for any formal comparative inference: at these sample sizes, Type II error (failure to detect a true class-specific effect) is near-certain, and the descriptive patterns in Supplementary Table S2 must therefore be interpreted solely as hypothesis-generating observations. No causal or comparative conclusions regarding individual antibiotic classes should be drawn from these data. The moderate overall sample size (*n* = 127) constrains event-per-variable ratios in regression models; null findings should therefore be interpreted as absence of large clinical effect, not as definitive proof of absence of effect. The absence of systematic microbiological culture data is a fundamental methodological constraint of this study: PCT served as a validated surrogate biomarker for bacterial co-infection probability, but biomarker surrogates cannot substitute for culture-confirmed microbiological diagnosis. Without culture data, definitive assessment of antibiotic appropriateness at the individual patient level is impossible—the stewardship analysis presented here characterizes prescribing behavior relative to biomarker-estimated bacterial probability, not relative to confirmed microbiological necessity. Future studies in this area must incorporate systematic blood and respiratory culture protocols, rapid molecular diagnostics, and resistance profiling to enable true antibiotic appropriateness assessment and pathogen characterization. Residual confounding by unmeasured variables—including corticosteroid dose and duration, antiviral therapy, concomitant immunosuppression, and nursing and physiotherapy intensity—cannot be excluded despite multivariable adjustment. In addition, although vaccination status was recorded as the number of doses received, prior vaccine-related adverse reactions were not systematically captured. Self-perceived overall health and health behavior variables, including smoking status, alcohol intake, and physical activity, were also not consistently available, which may have limited adjustment for baseline functional reserve and lifestyle-related confounding.

The post-COVID outcome assessment focused on respiratory and cardiac sequelae and did not include systematic evaluation of thromboembolic complications, which represent a separate and clinically significant component of the post-COVID syndrome [26]. Inclusion of structured thromboembolic surveillance is warranted in future cohort designs.

Heterogeneity across four pandemic waves encompassing the ancestral Wuhan strain through Delta and Omicron subvariants, with evolving therapeutic protocols including dexamethasone, remdesivir, tocilizumab, and baricitinib, introduces time-varying confounding that is difficult to fully account for in a single-cohort analytical framework. The predominantly unvaccinated cohort (61.4%) may limit direct applicability to post-Omicron populations with vaccine-modified disease severity and potentially different bacterial co-infection rates and microbiome profiles. The absence of gut microbiome profiling data precluded direct mechanistic assessment of antibiotic-induced dysbiosis at the molecular level, limiting conclusions on the gut–lung axis hypothesis to clinical outcome associations rather than causal mechanisms. The three-month follow-up horizon, while capturing the primary PASC assessment window, may not reflect longer-term trajectories at 12 or 24 months, particularly for CT pulmonary fibrosis, which may resolve or progress substantially over extended periods. Finally, the absence of a comparator arm treated under prospective PCT-guided stewardship prevents direct quantification of the clinical and AMR benefits that structured biomarker-guided protocols might confer in this setting. Multicenter prospective trials with systematic microbiological surveillance, gut microbiome profiling, and extended longitudinal follow-up are warranted to consolidate these preliminary findings and establish evidence-based antibiotic indications for COVID-19 and future viral respiratory pandemic preparedness.

## 5. Conclusions

In this cohort of 127 hospitalized COVID-19 patients, antibiotic therapy was prescribed to 53.5% with a paradoxical pattern of highest use in those with PCT ≤0.25 ng/mL (viral etiology likely), formally quantifying stewardship failure in a pandemic context. After multivariable adjustment, antibiotic use was not an independent predictor of 30-day mortality, ICU admission, or—in the post-COVID era—persistent dyspnea or pulmonary fibrosis at three months. COVID-19 disease severity was the sole independent determinant of both acute mortality and long-term respiratory sequelae. The composite severity score demonstrated excellent mortality discrimination (AUC = 0.953).

These findings carry a dual message for the post-COVID era. First, structured biomarker-guided antibiotic stewardship programs—incorporating PCT-based decision algorithms and rapid microbiological diagnostics—are needed for future epidemic respiratory disease management, both to limit AMR and with the confidence that reducing antibiotic use does not worsen long-term patient outcomes. Second, the high prevalence of post-COVID sequelae (persistent dyspnea 35.6%, CT pulmonary fibrosis 14.4%, reduced 6MWT in all survivors)—driven by viral severity rather than treatment decisions—highlights the need for systematic, severity-stratified long-term follow-up programs as a standard of care in the post-pandemic era. Multicenter prospective trials with systematic microbiological surveillance are warranted to consolidate these findings and establish evidence-based antibiotic indications for COVID-19 and future viral respiratory pandemics.

## Figures and Tables

**Table 1:** Baseline characteristics of the cohort stratified by antibiotic exposure.

Characteristics	Total Cohort (*n* = 127)	Antibiotic Group (*n* = 68)	No-Antibiotic Group (*n* = 59)
Demographics			
Age, years—median (IQR)	70.3 (64.0–80.6)	70.1 (64.0–80.4)	71.0 (63.7–81.5)
Male sex—*n* (%)	81 (63.8)	43 (63.2)	38 (64.4)
BMI, kg/m^2^—median (IQR)	27.3 (24.5–30.8)	26.5 (24.5–30.8)	27.8 (24.6–31.4)
Vaccination Status			
Unvaccinated—*n* (%)	78 (61.4)	43 (63.2)	35 (59.3)
One dose—*n* (%)	15 (11.8)	9 (13.2)	6 (10.2)
Two doses—*n* (%)	29 (22.8)	14 (20.6)	15 (25.4)
Three doses—*n* (%)	5 (3.9)	2 (2.9)	3 (5.1)
**COVID-19 Severity**			
Mild—*n* (%)	15 (11.8)	9 (13.2)	6 (10.2)
Moderate—*n* (%)	82 (64.6)	43 (63.2)	39 (66.1)
Severe—*n* (%)	24 (18.9)	11 (16.2)	13 (22.0)
Critical—*n* (%)	6 (4.7)	5 (7.4)	1 (1.7)
Comorbidities			
Hypertension—*n* (%)	72 (56.7)	39 (57.4)	33 (55.9)
Diabetes mellitus—*n* (%)	37 (29.1)	19 (27.9)	18 (30.5)
Obesity—*n* (%)	40 (31.5)	21 (30.9)	19 (32.2)
Heart failure—*n* (%)	30 (23.6)	22 (32.4) *	8 (13.6) *
Ischemic heart disease—*n* (%)	25 (19.7)	14 (20.6)	11 (18.6)
COPD/asthma—*n* (%)	19 (15.0)	12 (17.6)	7 (11.9)
Chronic kidney disease—*n* (%)	14 (11.0)	7 (10.3)	7 (11.9)
Vital Signs at Admission			
SBP, mmHg—median (IQR)	125.0 (114.0–138.5)	124.0 (113–135)	126.0 (115–140)
Heart rate, bpm—median (IQR)	96.0 (85.0–105.0)	96 (85–104)	96 (85–106)
Respiratory rate,/min—median (IQR)	24.0 (20.0–26.5)	24 (20–26)	24 (20–27)
SpO_2_, %—median (IQR)	92.0 (90.0–95.0)	91.0 (89–95)	93.0 (90–95)
Temperature, °C—median (IQR)	38.3 (37.7–38.8)	38.4 (37.8–38.8)	38.2 (37.7–38.8)
Laboratory Parameters			
CRP, mg/L—median (IQR)	87.6 (61.9–154.3)	84.1 (62.6–145.1)	90.4 (58.5–183.0)
Procalcitonin, ng/mL—median (IQR)	0.4 (0.2–0.6)	0.3 (0.2–0.6)	0.5 (0.2–0.7)
D-dimer, ng/mL—median (IQR)	802.0 (500.0–1387.5)	890.0 (591.5–1704.2) †	621.0 (404.5–1229.0) †
IL-6, pg/mL—median (IQR)	42.8 (15.0–90.2)	43.3 (14.8–87.8)	42.8 (15.1–92.2)
WBC, ×10^9^/L—median (IQR)	8.7 (5.7–10.8)	8.9 (5.4–10.7)	8.5 (5.8–11.2)
Lymphocytes, ×10^9^/L—median (IQR)	1.2 (0.8–1.7)	1.1 (0.8–1.5)	1.3 (0.9–1.8)
Creatinine, mg/dL—median (IQR)	1.1 (0.8–1.7)	1.1 (0.8–1.7)	1.1 (0.8–1.7)
Albumin, g/dL—median (IQR)	3.2 (2.9–3.6)	3.2 (2.9–3.5)	3.3 (3.0–3.7)
NT-proBNP, pg/mL—median (IQR)	506.0 (215.0–1145.5)	622.0 (263.0–1511.2)	438.0 (175.5–782.0)
SpO_2_/FiO_2_ ratio—median (IQR)	223.6 (163.0–276.4)	218.1 (164.6–277.8)	232.8 (162.6–274.1)
Severity score—median (IQR)	7 (4–11)	8 (5–12)	6 (4–10)

IQR = interquartile range; SBP = systolic blood pressure; CRP = C-reactive protein; WBCs = white blood cells; IL-6 = interleukin-6; NT-proBNP = N-terminal pro-brain natriuretic peptide; COPD = chronic obstructive pulmonary disease. * *p* = 0.020 (Fisher’s exact test); † *p* = 0.031 (Mann–Whitney U test). All other comparisons *p* > 0.05.

**Table 2:** Clinical outcomes and antibiotic prescribing details: antibiotic vs. no-antibiotic group.

Outcome	Antibiotic Group (*n* = 68)	No-Antibiotic Group (*n* = 59)	Test	*p* Value
Primary Endpoints				
30-day mortality—*n* (%)	13 (19.1)	10 (16.9)	Fisher	0.820
ICU admission—*n* (%)	15 (22.1)	10 (16.9)	Fisher	0.510
Mechanical ventilation—*n* (%)	10 (14.7)	4 (6.8)	Fisher	0.255
Secondary Endpoints				
Length of stay, days—median (IQR)	11.5 (9.0–15.5)	12.0 (10.0–15.0)	MWU	0.607
AKI (renal complications)—*n* (%)	16 (23.5)	15 (25.4)	Fisher	0.838
Cardiac complications—*n* (%)	18 (26.5)	12 (20.3)	Fisher	0.530
Sepsis—*n* (%)	4 (5.9)	5 (8.5)	Fisher	0.732
Sensitivity Analysis (Moderate–Critical, *n* = 112)				
30-day mortality—*n* (%)	13 (22.0)	10 (18.9)	Fisher	0.816
Length of stay—median (IQR)	12.0 (10–16)	13.0 (10–16)	MWU	0.838
Antibiotic Regimen Details				
Monotherapy—*n* (%)	50 (73.5)	—	—	—
Combination therapy—*n* (%)	18 (26.5)	—	—	—
Escalation—*n* (%)	12 (17.6)	—	—	—
Duration, days—median (IQR)	10 (7–14)	—	—	—
Antibiotic Indications (*n* = 68)				
Suspected bacterial co-infection	24 (35.3)	—	—	—
Bacterial pneumonia	16 (23.5)	—	—	—
Sepsis	15 (22.1)	—	—	—
Urinary tract infection	7 (10.3)	—	—	—
No documented indication	6 (8.8)	—	—	—

AKI = acute kidney injury; ICU = intensive care unit; IQR = interquartile range; MWU = Mann–Whitney U test. All *p* values are two-tailed; *p* < 0.05 was considered statistically significant. Sensitivity analysis restricted to moderate, severe, and critical cases confirmed null findings for primary endpoints.

**Table 3:** Multivariable logistic regression for 30-Day mortality and ICU admission.

Variable	30-Day Mortality OR (95% CI)	*p* Value	ICU Admission OR (95% CI) †	*p* Value
Antibiotic use	0.98 (0.27–4.05)	0.970	1.12 (0.31–4.05)	0.862
Age, per year	1.02 (0.99–1.08)	0.269	1.03 (0.99–1.08)	0.215
Male sex	1.34 (0.41–5.50)	0.741	1.28 (0.35–4.72)	0.712
COVID-19 severity *	3.56 (1.64–12.68)	0.018 **	—	—
COVID-19 severity (dichotomized) ‡	—	—	6.85 (2.12–22.14)	<0.001 **
D-dimer, log-transformed	0.97 (0.39–2.39)	0.942	1.05 (0.42–2.65)	0.912
Heart failure	1.72 (0.35–9.23)	0.611	3.45 (0.92–12.95)	0.066
IL-6, log-transformed	1.22 (0.72–2.59)	0.561	—	—

OR = odds ratio; CI = confidence interval. Multivariable logistic regression with Firth’s penalized maximum likelihood estimation; 95% CI derived from profile penalized likelihood. * Ordinal variable (mortality model): 0 = mild, 1 = moderate, 2 = severe, 3 = critical (WHO classification). † Odds ratios refer to the association between each predictor and ICU admission, which was analyzed as a secondary outcome in a separate logistic regression model. ‡ Dichotomized variable (ICU model): mild/moderate (*n* = 97) vs. severe/critical (*n* = 30); the ordinal severity variable was dichotomized to address quasi-complete separation arising from the small number of critical cases (*n* = 6). ** Statistically significant (*p* < 0.05). Antibiotic use was not an independent predictor of either outcome after adjustment for confounders. *n* = 127 for both models.

**Table 4:** Three-month follow-up outcomes in COVID-19 survivors.

Parameter	All Survivors (*n* = 104)	Antibiotic Group (*n* = 55)	No-Antibiotic Group (*n* = 49)
Mortality & Readmission			
Late death (post-discharge)—*n* (%)	4 (3.8)	2 (3.6)	2 (4.1)
Hospital readmissions—mean (SD)	0.28 (0.61)	0.29 (0.59)	0.27 (0.63)
Respiratory Outcomes			
Persistent dyspnea—*n* (%)	37 (35.6)	21 (38.2)	16 (32.7)
SpO_2_ at rest, %—median (IQR)	96.0 (93.0–98.0)	96.0 (93–98)	96.0 (94–98)
SpO_2_ on exertion, %—median (IQR)	93.0 (91.0–95.0)	93.0 (91–95)	93.5 (91–95)
CT pulmonary fibrosis—*n* (%)	15 (14.4)	9 (16.4)	6 (12.2)
Functional Capacity			
6-minute walk test, m—median (IQR)	335 (289–386)	329 (282–378)	359 (305–395)
NYHA functional class—median	2	2	2
Cardiac Biomarkers			
NT-proBNP, pg/mL—median (IQR)	296 (222–399)	296 (218–397)	296 (224–403)
Troponin, ng/mL—median (IQR)	0.007 (0.005–0.010)	0.007 (0.005–0.009)	0.007 (0.005–0.010)
LVEF, %—median (IQR)	58.0 (52–63)	57.0 (51–62)	59.0 (53–64)
Multivariable Analysis (Persistent Dyspnea) *			
Antibiotic use—OR (95% CI)	1.507 (0.624–4.162)	—	*p* = 0.391
COVID-19 severity—OR (95% CI)	1.226 (0.474–3.861)	—	*p* = 0.698
SpO_2_/FiO_2_ ratio—OR (95% CI)	1.001 (0.992–1.008)	—	*p* = 0.871

NYHA = New York Heart Association; CT = computed tomography; LVEF = left ventricular ejection fraction; NT-proBNP = N-terminal pro-brain natriuretic peptide; SD = standard deviation; IQR = interquartile range. No statistically significant between-group differences for any follow-up parameter (all *p* > 0.05, Fisher’s exact or Mann–Whitney U as appropriate). * Logistic regression with Firth’s penalized profile likelihood 95% CI; n = 104 survivors.

## Data Availability

Deidentified clinical, laboratory, and imaging data supporting the findings of this study are available from the corresponding authors upon reasonable request, subject to institutional data sharing policies.

## Supplementary Materials

The following supporting information can be downloaded at: https://www.mdpi.com/article/10.3390/microorganisms14061274/s1, Table S1: Distribution of patients across COVID-19 pandemic waves; Table S2: Clinical and follow-up outcomes stratified by antibiotic class.

## Abbreviations

The following abbreviations are used in this manuscript:

AKI	acute kidney injury
AMR	antimicrobial resistance
AUC	area under the curve
BMI	body mass index
CAP	community-acquired pneumonia
CI	confidence interval
COPD	chronic obstructive pulmonary disease
CRP	C-reactive protein
CT	computed tomography
FiO_2_	fraction of inspired oxygen
HAP	hospital-acquired pneumonia
HRCT	high-resolution computed tomography
ICU	intensive care unit
IL-6	interleukin-6
IQR	interquartile range
LVEF	left ventricular ejection fraction
MDR	multi-drug resistant
MRSA	methicillin-resistant *Staphylococcus aureus*
NT-proBNP	N-terminal pro-B-type natriuretic peptide
NYHA	New York Heart Association
OR	odds ratio
PASC	post-acute sequelae of SARS-CoV-2 infection
PCR	polymerase chain reaction
PCT	procalcitonin
ROC	receiver operating characteristic
SD	standard deviation
SpO_2_	peripheral oxygen saturation
UTI	urinary tract infection
WBC	white blood cell count
WHO	World Health Organization
6MWT	six-minute walk test
