# Supplementary material for: Antibiotic Use Patterns and Clinical Outcomes in Hospitalized COVID-19 Patients: A Single-Center Observational Cohort Study with Three-Month Follow-Up
Source: Microorganisms. 2026 Jun 5;14(6):1274. doi: 10.3390/microorganisms14061274 (PMC13304473; doi:10.3390/microorganisms14061274)
Supplement: Supplementary file 1 [file microorganisms-14-01274-s001.zip › microorganisms-4311657-supplementary.pdf]

Supplementary Materials

Supplementary Table S1. Distribution of patients across COVID-19 pandemic waves.

Wave	Period	Dominant Variant	n (%)	Antibiotic use n (%)	30-day mortality n (%)
1	Mar 2020 – Jun 2021	Ancestral/Alpha	42 (33.1)	28 (66.7)	12 (28.6)
2	Jul 2021 – Dec 2021	Delta	35 (27.6)	22 (62.9)	8 (22.9)
3	Jan 2022 – Jun 2022	Omicron BA.1/BA.2	28 (22.0)	12 (42.9)	2 (7.1)
4	Jul 2022 – Dec 2024	Omicron BA.5 and subsequent	22 (17.3)	6 (27.3)	1 (4.5)
Total	—	—	127 (100)	68 (53.5)	23 (18.1)

Supplementary Table S2. Clinical and Follow-Up Outcomes Stratified by Antibiotic Class.

Antibiotic Class	n	30-day Mortality n (%)	ICU Admission n (%)	LOS, days Median (IQR)	Persistent Dyspnea at 3M n (%)	CT Fibrosis at 3M n (%)	Indication (typical)
No Antibiotic	59	10 (16.9)	10 (16.9)	12 (10–15)	16 (27.1)	6 (10.2)	—
Glycopeptide	21	5 (23.8)	5 (23.8)	12 (10–15)	5 (23.8)	2 (9.5)	MRSA/GPC
Macrolide	12	1 (8.3)	1 (8.3)	10 (6–13)	5 (41.7)	1 (8.3)	Atypical
Fluoroquinolone	10	1 (10.0)	2 (20.0)	10 (9–14)	2 (20.0)	2 (20.0)	CAP/UTI

Carbapenem	9	3 (33.3)	3 (33.3)	13 (12–18)	4 (44.4)	1 (11.1)	Sepsis/MDR
Beta-lactam	10	2 (20.0)	4 (40.0)	12 (9–24)	1 (10.0)	2 (20.0)	CAP/HAP
Cephalosporin	6	1 (16.7)	0 (0.0)	10 (9–13)	4 (66.7)	1 (16.7)	CAP/UTI

LOS = length of hospital stay; ICU = intensive care unit; CT = computed tomography; 3M = 3-month follow-up; CAP = community-acquired pneumonia; HAP = hospital-acquired pneumonia; UTI = urinary tract infection; MDR = multi-drug resistant organism; MRSA = meticillin-resistant Staphylococcus aureus; GPC = gram-positive cocci. Formal inter-class comparisons were not performed due to small subgroup sizes. Indication column reflects predominant documented clinical rationale.
